# Analysis of the changes in quality and characteristics of hot air drying of Xinjiang jujube (*Zizyphus jujuba Mill*. *cv. Junzao*) following a delayed harvest

**DOI:** 10.1038/s41598-023-43594-w

**Published:** 2023-10-04

**Authors:** Chao Xu, Xiaokang Yi, Can Hu, Qiaonan Yang, Jie Li, Jie Zhang, Yi Yang

**Affiliations:** 1 College of Mechanical and Electrochemical Engineering, Tarim University, Arar, 843300 China; 2 College of Mechanical and Electrical Engineering, Fujian Agricuture and Forestry University, Fuzhou, 350000 China

**Keywords:** Plant sciences, Plant physiology

## Abstract

Dry processing is ineffective in preserving fresh jujubes (*Zizyphus jujuba* Mill.), contributing largely to the delayed jujube harvest in Xinjiang. However, no studies have evaluated the impact of delayed harvest periods on processing quality. Therefore, the present study investigated the effects of different delayed harvest periods on the characteristics of the quality of jujubes in Xinjiang after hot air drying. Six batches (S1–S6) were sampled over a 7-d period. Various indicators of jujubes changed significantly during the extended harvest period (*P* < 0.05). The water content of the fruit decreased progressively. While the percentages of soluble solids, total sugars, and reducing sugars increased continuously, the total weight of these parameters in a single jujube fruit decreased continuously. The proportion of ascorbic acid, total weight, and drying time decreased steadily. The fruit had the highest ascorbic acid content at the S4 stage after hot air drying (87.14 mg 100 g^−1^). Fewer color differences were recorded in hot air-dried fruits as compared with fresh jujubes; the cracking rate decreased after hot air drying, but the fruit could be rehydrated more effectively. A comprehensive evaluation revealed that jujubes harvested in the S4 stage were better suited for dry processing.

## Introduction

China, being the origin of jujube (*Zizyphus jujuba* Mill.), produces more than 95% of the world's total amount of jujubes grown each year^1^. Xinjiang is the largest producer of jujubes in China, with an annual production of nearly 4 million tons owing to its unique geographical and climatic conditions^2^. This accounts for more than 45% of the overall output in China^3^ and is primarily composed of the Junzao cultivar and gray jujube. The combined output of the two accounts for more than 90% of the annual jujube production in Xinjiang^4^. The jujube fruit is large, nutritious, sweet and sour, rich in sugars, organic acids, ascorbic acids, phenolics, and other nutrients^5^. Fresh jujubes are fermentable due to their high water content and often lose up to 15–30% of their value after harvesting^6^. Therefore, 95% of jujubes are dried and sold worldwide^7^. Drying technology for jujube is currently established, with typical drying methods, including hot air^8^, microwave^9^, vacuum freezing^10^, and infrared drying^11^. Hot air drying has become the primary method for jujube drying in Xinjiang, China, because it does not require specialized equipment, is simple to set up, consumes less energy, and is highly efficient^12^. Xinjiang jujube is not harvested immediately when ripe. They are dried in the sun, wind, or other natural forces to reduce the water content within the jujube; this process, known as delayed harvest, lasts approximately 40 days. The approach of determining the delayed jujube harvest is based primarily on farmer experience and market demand, and it does not consider the influence on jujube quality, which has yet to be validated.

()Many researchers have investigated how fruit quality changes with growth stage^13^. Gou Xi et al.^14^ examined variations in the contents of cyclic adenosine phosphate, soluble sugar (SS), and mineral element of Lingwu jujube at different stages of maturity. Zhang Kekun et al.^15^ found significant differences in the characteristics of grape (*Vitis vinifera* L.) quality at different stages of hang-on tree storage. Deng et al.^16^ investigated the physicochemical parameters and drying kinetics of apricot (*Prunus armeniaca* L.) fruit at different maturity stages and discovered that the drying rate decreased after a particular stage of maturity. Liu Qinyuan et al.^17^ revealed that the aroma and color of the finished product differed when peaches (*Pr. persica* [L.] Batsch) at different levels of maturity were used to manufacture wine. Wang et al.^18^ evaluated the effects of postharvest ripening on the physicochemical properties, microstructure, cell wall polysaccharide content, and nanostructure of kiwifruit (*Actinidia deliciosa* [A. Chev] CF Liang et AR Ferguson). Tang Ni et al.^19^ found that the crude fat and contents of 17 amino acids of different avocado varieties (*Persea americana* Mill.) achieved peak levels. The optimal harvest period for each avocado variety was determined by monitoring changes in the curve of the content of the components. Hu Can et al.^20^ investigated the variations in quality and drying kinetics between delayed harvesting and drying of apricots in Xinjiang. Diop Alioune et al.^21^ assessed drying mangoes (*Mangifera indica* Linn.) and discovered that the fruit browning was more pronounced as the mangoes ripened; these data demonstrated that ripeness significantly influenced the color of dried mangoes. While the above data strongly demonstrate that the physical and chemical properties and processing characteristics of fruit differ significantly at different stages of harvesting, to our knowledge, no investigations have been reported on potential changes in quality over the jujube drying period.

The present study investigated the effect of different picking stages on the physicochemical and drying characteristics of jujubes. The process from ripening to actual harvest was categorized into six sampling stages, and the content and changes in total sugars (TS) and reducing sugars (RS), SS, titratable acids (TA), and ascorbic acid were analyzed. The drying characteristics of jujube at different picking stages and the retention of nutrient, color, and cracking rate after drying were analyzed using hot air drying to provide a theoretical reference for determining the optimal picking period suitable for drying and processing of jujube.

## Materials and methods

### Test materials

**Figure 1:**
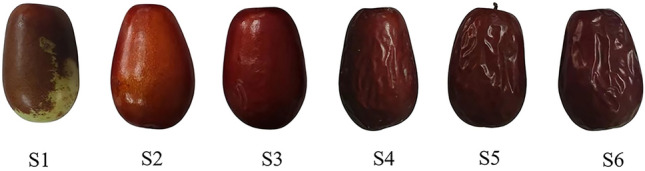
Photographs of jujubes at different picking stages in this experiment.

The jujube used in the experiment were harvested from farm No. 8, 10th regiment, Alar City, Xinjiang Uygur Autonomous Region, China (81°12′15.53″E, 40°38′16.21″N), 1,023 m a.s.l. Branches with the same orientation and fruit maturity were selected from 30 jujube trees that were 10 years old and had grown uniformly. Jujubes on the branches were marked (as designated by the Xinjiang Special Forestry and Fruit Industry Export Marketing Board^22^. The first picking was performed on September 16, 2022, with a 7-d interval for each sample. Six batches of samples, designated S1–S6, were collected. The samples were cleaned with alcohol, sealed, and stored at 4 °C to prevent the loss of moisture and nutrients. Batches of experiments were conducted the next day to minimize errors that occur when sampling the fruit over a longer period. Table 1 depicts the surface conditions of jujubes at different picking stages. Figure 1 depicts the photographs of jujubes at different picking stages in this experiment.

**Table 1:** Sensory characteristics of jujubes at different picking stages.

Picking stage	Picking date	Surface condition
S1	16 September	Reddening of the surface over 75%, bright color, and full and firm fruit
S2	24 September	Over 95% of the surface reddened, bright color and full and firm of fruit
S3	1 October	Surface all red, brighter in color, surface ruffled
S4	8 October	Surface all red, duller in color, with increased surface ruffling
S5	16 October	Surface all red, dull, with distinctive surface folds
S6	23 October	Surface all red, dull, with distinctive surface folds

The experimental site was selected from the key Laboratory of Modern Agricultural Engineering, Tarim University, Alar, Xinjiang Uygur Autonomous Region, and the experiment ran from September 16, 2022 to October 30, 2022. All experiments followed the relevant national, international, or institutional guidelines and regulations.

### Measurement methods for each indicator

#### Determination of moisture content and average weight

The weight and moisture content were determined as described by Pu^23^.

#### Determination of total sugars and reducing sugars

The TS content was determined as described in GB/T 10782-2021, "General rules for the quality of preserves"^24^. The RS content was determined using the direct titration method in GB5009.7-2016 "Determination of reducing sugars in foodstuffs"^25^.

#### Determination of the titratable acids

Ten jujubes were selected, and 15 g of flesh was randomly weighed from different parts of the fruit and crushed. The TA was determined from the collected filtrate by neutralization titration with sodium hydroxide^26^. Malic acid was the primary acid in the jujube^27^.

#### Determination of the soluble solids (SSC)

Ten jujubes were selected, and 5 g of samples were randomly weighed from various parts of different jujubes and pulverized. The filtrate was collected and deposited in the test area of a hand-held refractometer (LH-Y28, Lu Heng China) for measurement and reading at 22 °C. The results were expressed as a percentage of the total mass fraction.

#### Determination of ascorbic acid (VC) content

Ascorbic acid content was determined using the 2,6-dichloroindophenol titration method described in GB 5009.86-2016^28^.

### Determination of color

The color variation between picked and dried jujubes from S1 to S6 was determined using a spectrophotometer (YS6003, Sanenshi China) and the CIELAB color chart system. The brightness L*, green–red value a*, and blue-yellow value b* were determined. The color difference in values between the colors at the different picking stages and the jujubes dried in hot air were analyzed and calculated as follows^29^:

1 $$\Delta {\text{E}} = \left[ {\left( {\Delta {\text{L}}*} \right)^{{2}} + \left( {\Delta {\text{a}}*} \right)^{{2}} + \left( {\Delta {\text{b}}*} \right)} \right]^{{{1}/{2}}}$$

where ∆E denotes the value between different colors; ∆L*, ∆a*, and ∆b* denote the difference in color parameters between the fruit picked at stages S2–S6 and S1, respectively.

### Drying of jujubes

#### Drying equipment

Twenty jujubes of roughly the same size, shape, and hardness were selected from each batch, removed from 4 °C storage, brought to room temperature, and wiped dry. The starting weight was the whole weight of the fruit. The drying conditions were set at 65 °C, 1.5 m s^−1^ wind speed, and 40% relative humidity using a hot air thin layer drying test apparatus (homemade by the Key Laboratory of Modern Agricultural Engineering, Tarim University)^30^.

The dryer was switched on, and the heating and air supply device was initiated during the trial. After 15 min of operation, the material bin was preheated to set parameters and attain a constant value. The jujubes were then evenly distributed in the drying chamber trays. The weight sensor within the material bin was automatically weighed and recorded every hour. Tray quality was evaluated to determine their real-time moisture content until the dry base of the jujubes reached < 33.3%. The drying test was completed at this point. The fruit was left to cool, and the surface cracks were examined using a stereo microscope. The jujubes were bagged, sealed under a vacuum, and stored at room temperature before testing.

#### Drying parameters

(1) Moisture content of the dry base

The moisture content of jujubes varies as they dry. ${M}_{t}$ denotes the dry basis moisture content of jujubes at time t, and it is expressed as follows:

2 $${M}_{t}=\frac{{G}_{t}-{G}_{0}(1-{M}_{0})}{{G}_{0}(1-{M}_{0})}$$

where ${G}_{t}$ denotes the total weight of all jujubes in the material tray at time t (g). ${G}_{0}$ denotes the starting weight of all jujubes in the material tray (g), and ${M}_{0}$ denotes the initial wet base moisture content of the jujubes (%).

*(2) Drying rate*

The drying rate of any stage (Drying rate) is expressed as a function of the moisture content of the dry base at the start and end stages versus time as follows^31^:

3 $$DR=\frac{{M}_{{t}_{2}}-{M}_{{t}_{1}}}{{t}_{2}-{t}_{1}}$$

where ${\mathrm{M}}_{{\mathrm{t}}_{1}}$ denotes the dry basis moisture content at the start of the stage. ${\mathrm{M}}_{{\mathrm{t}}_{2}}$ denotes the dry basis moisture content at the end of the stage. ${\mathrm{t}}_{1}$ denotes the start of the phase, and ${\mathrm{t}}_{2}$ represents the end of the stage.

(3) Effective diffusion coefficient of moisture

The effective diffusion moisture coefficient during hot air drying of jujubes was calculated according to Fick's second law equation^32^:

4 $${\text{MR}} = \frac{6}{{{\uppi }^{2} }}\mathop \sum \limits_{{{\text{n}} = 1}}^{1} {\text{exp}}\left( { - \frac{{{\text{n}}^{2} {\uppi }^{2} {\text{D}}_{{{\text{eff}}}} }}{{{\text{r}}_{{\text{e}}}^{2} }}{\text{t}}} \right)$$

Where MR represents moisture content ratio; ${\mathrm{D}}_{\mathrm{eff}}$ denotes effective moisture diffusion coefficient (m^2^ s^−1^); r_e_ denotes volume equivalent radius (m), and t denotes drying time (s).

Simultaneously taking the logarithm of both sides of the equation yields the following:

5 $$\mathrm{lnMR}=\mathrm{ln}\frac{6}{{\uppi }^{2}}-\frac{{\uppi }^{2}{\mathrm{D}}_{\mathrm{eff}}}{{\mathrm{r}}_{\mathrm{e}}^{2}}\mathrm{t}$$

The slope method was used in calculating the effective diffusion coefficient of moisture. The slope expression from Eq. (5) is as follows:

6 $$\mathrm{k}=-\frac{{\uppi }^{2}{\mathrm{D}}_{\mathrm{eff}}}{{\mathrm{r}}_{\mathrm{e}}^{2}}$$

#### Observation of the superficial surface cracks

The high heat and mass transfer rate during jujube hot air drying accelerates heat expansion, water loss, and shrinking, which readily cause cracks. The presence of cracks results in nutrient loss, an enrichment of the microbial population and their entry into the fruit, and other conditions that influence drying quality. Each batch included 20 jujubes without initial cracks selected for drying. The surface of the jujubes was examined using a stereomicroscope (TD-2KH, Sanmang Teda China), and the rates of jujube cracking at different picking stages after hot air drying were calculated using the statistical method that is employed to calculate the cracks that occur in hazelnut (*Corylus* spp.) after drying^33^.

7 $$\mathrm{P}=\frac{{\mathrm{N}}_{\mathrm{d}}}{{\mathrm{N}}_{\mathrm{w}}}$$

where P denotes the rate of cracked fruits; ${\mathrm{N}}_{\mathrm{d}}$ denotes the number of stepped jujubes with cracks after hot air drying, and ${\mathrm{N}}_{\mathrm{w}}$ denotes the total number of dried jujubes.

#### Determination of the rehydration ratio

Rehydration is a crucial indicator of dried jujube quality. Five dried jujube were randomly selected, weighed, and placed at 50 °C for 40 min, dried on absorbent paper, and left to dry for 20 min and re-weighed. The rate of rehydration of jujubes was calculated as follows^34^:

8 $$\mathrm{R}=\frac{{\mathrm{M}}_{\mathrm{w}}}{{\mathrm{M}}_{\mathrm{d}}}$$

where R denotes the rehydration ratio; ${\mathrm{M}}_{\mathrm{d}}$ denotes the initial weight, and ${\mathrm{M}}_{\mathrm{w}}$ denotes the weight after rehydration.

### Integrated methods of evaluation

The Analytical Hierarchy Process (AHP) was used on a scale of 1–9. A pairwise comparison matrix of the factors was constructed, followed by a consistency test. The indicators used to evaluate the jujubes after hot air drying required normalization. The positive indicators included ascorbic acid content and rehydration ratio after hot air drying, whereas the negative indicators included drying time, cracking rate, and color difference. The normalization formula ${y}_{i}$ is illustrated in Eqs. (9) and (10):

9 $${y}_{i}=\frac{{x}_{i}-{x}_{min}}{{x}_{max}-{x}_{min}}$$

10 $${y}_{i}=\frac{{x}_{max}-{x}_{i}}{{x}_{max}-{x}_{min}}$$

where ${y}_{i}$ denotes the normalized values of the indicators; ${x}_{i}$ denotes the actual values of the indicators, and the ${x}_{max}$ and ${x}_{min}$ denotes the maximum and minimum values of the indicators, respectively.

The combined score for the drying quality of jujubes at different picking stages $\mathrm{y}$ was determined as follows:

11 $${\text{y}} = {\text{y}}_{1} {\text{l}}_{1} + {\text{y}}_{2} {\text{l}}_{2} + {\text{y}}_{3} {\text{l}}_{3} + {\text{y}}_{4} {\text{l}}_{4} + {\text{y}}_{5} {\text{l}}_{5}$$

where ${\mathrm{y}}_{1}$ ,${\mathrm{y}}_{2}$ ,${\mathrm{y}}_{3}$ ,${\mathrm{y}}_{4}$ and ${\mathrm{y}}_{5}$ are the normalized values for ascorbic acid content, rehydration ratio, drying time, cracking rate, and color difference, respectively, and ${\mathrm{l}}_{1}$ ,${\mathrm{l}}_{2}$ ,${\mathrm{l}}_{3}$ ,${\mathrm{l}}_{4}$ and ${\mathrm{l}}_{5}$ represent the corresponding weights.

### Statistical analysis

The data are expressed as mean ± SD. The data were subjected to a one-way analysis of variance (ANOVA) using SPSS 25.0 (IBM, Inc., Armonk, NY, USA) to examine significant differences in the median fruit index at different harvest periods (*P* < 0.05). The correlation of fruit quality indices was analyzed using Pearson's method. Furthermore, the correlation Plot plugin in Origin 2018 (OriginLab, Northampton, MA, USA) was employed to generate a correlation heat map of quality indicators. The consistency of the matrix was assessed using MATLAB.

## Results and discussion

### Moisture content and weight change analysis

**Figure 2:**
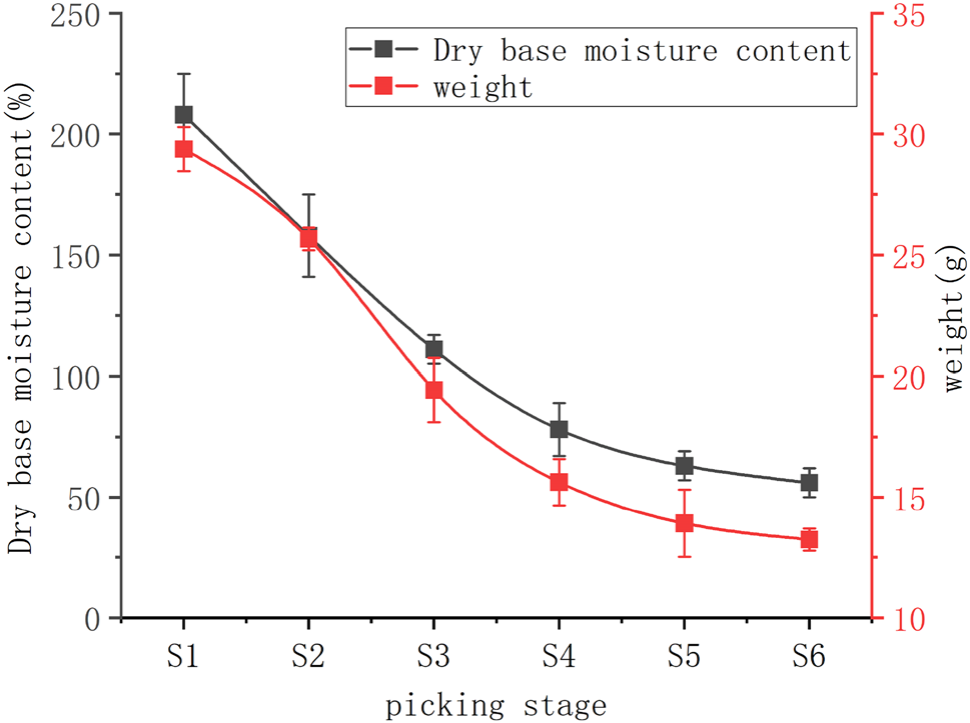
Graph of moisture content and weight change.

The moisture content and weight are the key parameters used to evaluate the growth state of jujubes^35^. As shown in Fig. 2, the dry basis moisture content and mean weight of jujubes picked at different stages of the delayed harvest period ranged from 208 to 56% and 13.32–29.38 g, respectively. The jujubes were dehydrated during the delayed picking period with significant differences (*P* < 0.05) between the moisture content and mean weight of those picked at S1–S4. The most significant reduction in water content was observed in stages S2–S3 and S3–S4. The fruit in this ripening period incurred the primary amount of dehydration during the delayed harvest period, compared with those harvested at stages S1–S2. There was less variation in moisture content, and the jujubes weighed less during the S1–S2 stages. These changes potentially occurred because the branches continually supply water and other materials to the fruit^36^. The range of change in moisture content in the S4-S6 stage decreased significantly (*P* < 0.05). The surface of the jujube lost water rapidly during this period, and natural environmental factors were insufficient for the internal water of the jujube to rapidly migrate to the surface and diffuse into the air^37^.

### Changes in the contents of nutrients

The total weight of material within a single jujube fruit was used to reveal the changes in the indicators during the delayed harvest periods as the moisture content and average weight of the batches of jujubes continued to decrease.

**Figure 3:**
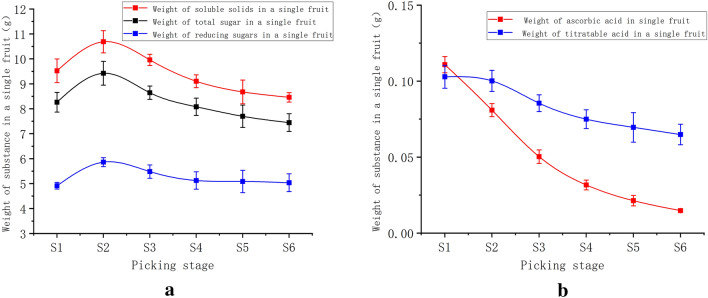
Variation in the total content of individual jujube fruits at each stage of delayed harvest.

SS represents solid substances that are water soluble in fruits, including sugars, acids, and trace elements. Sugar is the most essential nutrient and flavoring element in jujube^38^. As depicted in Table 2 and Fig. 3, the TS and RS ranges of variation were 28.13–56.22% and 16.73–38.02%, respectively, while the SS range was 32.41–63.89%. The percentage content of both the TS and RS increased as the harvest time was extended, possibly due to the evaporation of water in the fruit, resulting in a further concentration of dry matter. Furthermore, the total content within a single fruit increased and decreased, peaking at the S2 stage. The percentage TS content and total content of a jujube within a single fruit showed the highest increase from S1 to S2. Jujube is considered to be in the "reddening stage" and starch is metabolized to SS^39^. Simultaneously, the total content of compounds in a single jujube decreased at the S2–S6 stages, indicating that the jujubes absorbed internal nutrients during respiration. Fruit metabolism is reduced following a drop in its moisture content, and the loss rate of total nutritional content at each stage similarly decreases with the steady decrease in respiration^40^**.**

**Table 2:** Variation in the content of jujube components at various stages of delayed harvest.

Picking stage	Soluble solids %	Ascorbic acid mg/100 g	Total sugar %	Reducing sugar %	Titratable acid %
S1	32.41 ± 1.84e	377.25 ± 6.58a	28.13 ± 1.77d	16.73 ± 1.12d	0.35 ± 0.07b
S2	41.64 ± 2.77d	315.21 ± 10.81b	36.71 ± 2.54c	22.84 ± 1.59c	0.39 ± 0.02ab
S3	51.26 ± 0.45c	258.76 ± 5.32c	44.48 ± 0.85bc	28.22 ± 0.04bc	0.44 ± 0.05ab
S4	58.28 ± 1.21b	202.65 ± 8.04d	51.73 ± 2.01ab	32.81 ± 0.84ab	0.48 ± 0.01a
S5	62.37 ± 1.36a	153.43 ± 9.36e	55.34 ± 1.65a	36.56 ± 1.01a	0.50 ± 0.02a
S6	63.89 ± 2.15a	111.61 ± 4.94f.	56.22 ± 3.63a	38.02 ± 0.45a	0.47 ± 0.11ab

Ascorbic acid (Vitamin C) is an essential vitamin that the human body cannot synthesize^41^. The percentage of ascorbic acid varied from 377.25 mg 100 g^−1^ to 111.61 mg 100 g^−1^ during delayed harvest, following the same trend as the total content, and demonstrated a distinct declining trend during the S1-S6 stages, as previously described^42^. A hypothesis exists that the continuous loss of ascorbic acid during ripening and drying is related to its high susceptibility to oxidation.

TA can significantly change the taste of fruit in general^43^. The level of total TA in a single jujube fruit increased slightly from S1 to S2 throughout the delayed harvest period. The total content of jujube gradually decreased during the S2–S6 stage. Minyan^44^ et al. also discovered that the titrable acid content of jujube decreased with maturity during the jujube growing phase. As a result, jujube was sweeter and less sour after delayed harvesting, which explains why we selected it as the main raw material for Xinjiang dried fruit processing.

### Color changes

The appearance and color of the fruit is the primary determinant of consumer purchase^45^. Table 3 outlines the change in color at the delayed harvest stage.

**Table 3:** Color changes in jujubes at different stages of delayed harvest.

Picking stage	Brightness L*	Red and green values a*	Blue and yellow values b*	Color difference value ΔE
S1	51.26 ± 5.64a	12.75 ± 3.91b	35.54 ± 2.15a	–
S2	39.82 ± 1.29b	20.13 ± 2.58a	22.12 ± 0.55b	16.00 ± 1.21c
S3	34.58 ± 0.88c	22.21 ± 1.54a	16.35 ± 1.72c	25.09 ± 2.54b
S4	31.48 ± 0.97d	21.92 ± 2.21a	14.03 ± 0.36d	30.62 ± 2.25a
S5	31.51 ± 1.04d	22.45 ± 2.53a	14.01 ± 1.13d	30.78 ± 2.49a
S6	29.53 ± 1.26d	23.28 ± 0.74a	13.65 ± 1.11d	32.59 ± 0.77a

The brightness L* of jujubes decreased significantly (*P* < 0.05) throughout the delayed harvest process in the S1–S4 stage. The S1–S2 stage decreased dramatically from 51.26 to 39.82 due to the green coloration on the epidermis of the S1 stage fruit. The contrast between green and red increased. The brightness in the S2–S4 stage dropped from 39.82 to 31.48 because of the rapid water loss between these stages. The light reflection decreased parallel with water loss from the epidermal cells, and the fruit turned from a luminous maroon to a dull dark red. However, there was no statistically significant change in brightness between the S4 and S6 stages (*P* < 0.05).

The surface condition of jujubes progressively stabilized after the S4 stage. The red-green value a* was significantly lower in the S1 stage than in the S2–S6 stages. The red-green value a* did not change significantly after the S2 stage, which was consistent with the S1 stage. The "red-change" stage is considered the most critical for changes in the compounds responsible for fruit coloring^46^. The blue-yellow value b* decreased significantly between S1 and S4 and did not change significantly between S4 and S6, which is consistent with the trend of brightness L*. The chromatic aberration value of the S2–S4 stages increased and stabilized during the S4-S6 stage such that the chromatic aberration value of each stage of S1-S4 was significant. However, there was no significant difference in the S4–S6 stage and these results may be because water content influences the material coloration; water on the surface of the jujube is completely lost after the S4 stage, and the color change remains insignificant. Thus, while differences in fruit coloration might indicate jujube maturity in the growth phase^47^, they cannot be used as a criterion for the delayed harvest procedure.

### Quality correlation analysis

**Figure 4:**
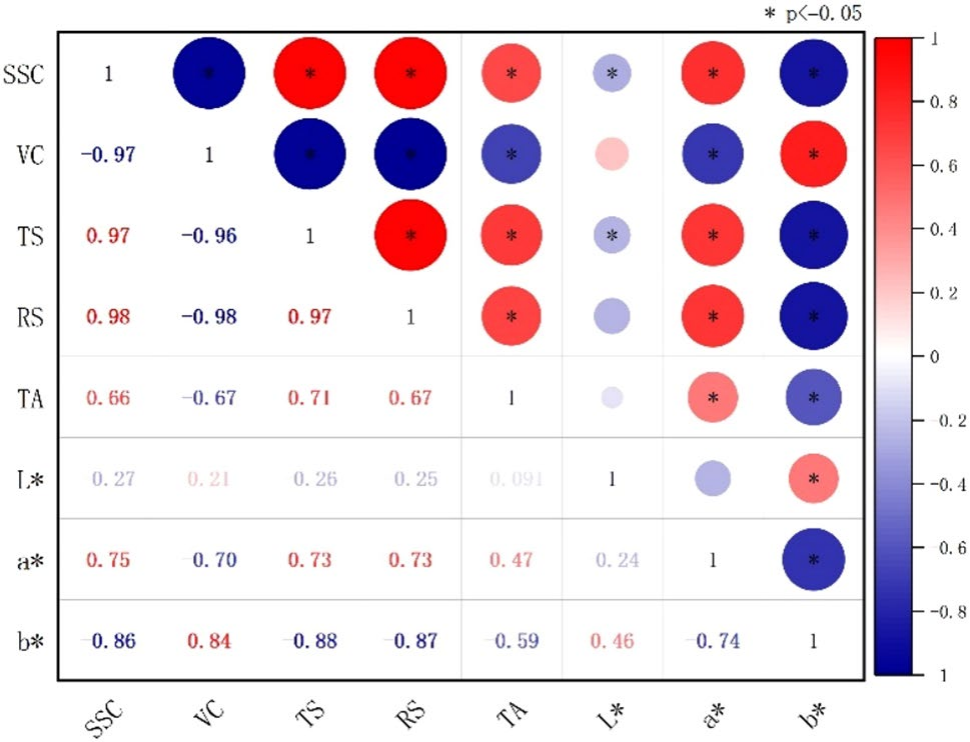
Correlation coefficient diagram showing the quality of jujubes at different stages of delayed harvest. Based on Pearson's correlation analysis, red represents positive correlation, blue represents negative correlation, darker colors represent higher correlations, * indicates 0.05 level, and the numbers in the graph indicate correlation coefficients between indicators.

The correlation coefficient between the jujube quality indices at the different stages of delayed harvest is depicted in Fig. 4, and the SS content significantly positively correlated with the TS, RS, TA, red and green values (*P* < 0.05), and ascorbic acid and blue-yellow values (*P* < 0.05). Ascorbic acid positively correlated with the TS, RS, red-green values, and TA (*P* < 0.05), and negatively with the blue-yellow values (*P* < 0.05). The TS and RS, TA, and red-green values all had a significant positive correlation, whereas the blue-yellow values had a significant negative correlation (*P* < 0.05). The red-green and blue-yellow values had a significant negative correlation (*P* < 0.05). In a nutshell, changes in various indicators are co-related rather than independent, exhibiting positive or negative correlations during the delayed harvesting process. The SS comprised soluble sugars, acids, minerals, and other water-soluble compounds, and the SS accounted for the largest proportion. As the harvesting process was delayed, the moisture content of jujubes decreased, while the percentage content of SS, TS, RS, and TA increased. However, the unstable nature of VC increased the rate of loss on the trees throughout the drying process. As a result, VC was a negative correlation with the other nutritional indicators. Brightness and indicators were weakly correlated. There was a high correlation between the blue-yellow and red-green values for the other indicators. Thus, the blue-yellow and red-green values indicate the delayed harvest stages.

### Drying characteristics

#### Influence of the picking stage on the kinetics of hot air drying of jujubes

**Figure 5:**
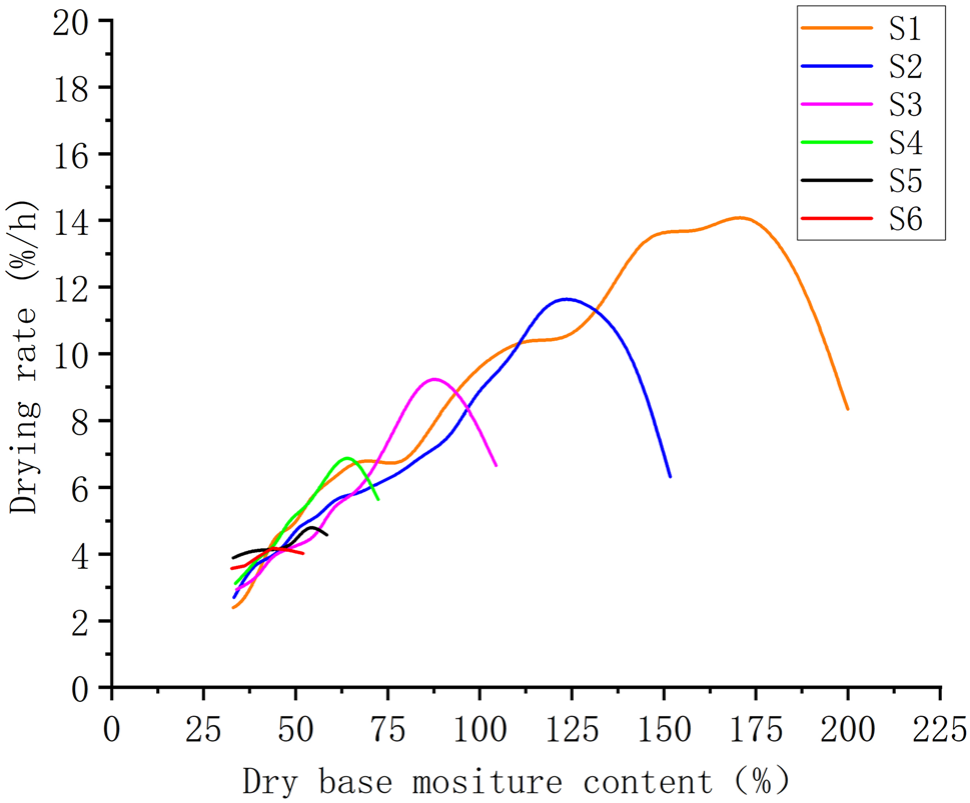
Hot air drying rate curves for different stages of delayed harvest.

Figure 5 depicts the hot air drying rate curves for the different picking stages. The drying time required for each batch of jujubes from S1 to S6 was 22 h, 19 h, 14 h, 10 h, 7 h, and 6 h, respectively. Thus, the overall time for hot air drying decreased as the jujubes from the delayed harvest matured naturally, and the rate of drying of the samples from S1-S4 exhibited an increasing trend and then decreased. The jujubes were in the preheating and initial heating stage at the start of the drying period, and the drying rate increased first. The internal regulation of moisture diffusion decreased at a later stage^48^. The drying curves of the samples in S5–S6 were more comparable to those of uniform drying, indicating that lowering the initial moisture content hastened the preheating and heating process of the jujubes. It was easier to disperse moisture from the surface of the jujube during hot air drying than it was to move moisture from the interior of the jujube to the surface. However, at the end of drying, the drying rate in the S5–S6 stage was higher than at the S1–S4 stage.

Along with the characteristics of shrinkage of the porous media that holds moisture during drying, the high initial moisture content of jujube is regarded to be more destructive to the tissue structure during hot air drying due to the long drying period and quick rate of mass transfer. Furthermore, the drying rate inside the early drying stage is substantially lower than that on the surface of the jujube. Uneven water migration produces biological stress, causing the jujube to shrink, deform, and eventually destroy the water migration channel. Shrinkage of jujubes with a low initial moisture content is not readily visible due to slower heat and mass transfer, better preservation of the pore structure of the flesh, and increased efficiency of water passage during delayed harvest periods.

#### Effect of the picking stage on the effective diffusion coefficient of water in jujubes

The effective moisture diffusion coefficient reflects how rapidly moisture inside the material migrates to the surface during the drying process, which is influenced by the changes in material structure, moisture content, and drying temperature. The results are shown in Table 4. The effective moisture diffusion coefficient ranged from 9.3621 × 10^−10^ m^2^ s^−1^ to 10.7400 × 10^−10^ m^2^ s^−1^ from S1 to S6, exhibiting an increasing trend followed by a decreasing trend^43^. These data suggest that jujubes picked in the early stages have a high initial moisture content and rapid water diffusion during the initial drying stage; on the other hand, they produce severely wrinkled and crusted fruit, which distorts and damages the internal water transfer channels, reducing water diffusion. In contrast, jujubes picked too late exhibited a comparably slow water diffusion mechanism owing to their low initial moisture content and water primarily bonded internally. Jujubes e picked at S4 during the delayed harvest process had the highest effective water diffusion coefficients due to variations in moisture content and cracking.

**Table 4:** Variation in the effective diffusion coefficient of jujube moisture at different stages of delayed harvest.

Picking stage	Initial dry basis moisture content	Drying time h	Linear regression fitting formula	Decision factor R^2^	Effective diffusion coefficient of moisture ${D}_{eff}/(\times {10}^{-10}){m}^{2}\cdot {s}^{-1}$
S1	2.08 ± 0.17a	22	$\mathrm{lnMR}=-2.31{\times 10}^{-5}\mathrm{t}-0.1355$	0.9855	9.3621
S2	1.58 ± 0.17b	19	$\mathrm{lnMR}=-2.34{\times 10}^{-5}\mathrm{t}+$ 0.04423	0.9863	9.4837
S3	1.11 ± 0.06c	14	$\mathrm{lnMR}=-2.38{\times 10}^{-5}\mathrm{t}+0.0018$	0.9849	9.6458
S4	0.78 ± 0.11d	10	$\mathrm{lnMR}=-2.65{\times 10}^{-5}\mathrm{t}-0.0056$	0.9880	10.7400
S5	0.63 ± 0.06de	7	$\mathrm{lnMR}=-2.55{\times 10}^{-5}\mathrm{t}-0.0174$	0.9991	10.3348
S6	0.56 ± 0.06e	6	$\mathrm{lnMR}=-2.52{\times 10}^{-5}\mathrm{t}-0.0147$	0.9994	10.2132

### Drying quality of jujube at different picking stages

#### Cracking rate of jujube after hot air drying at different stages

**Figure 6:**
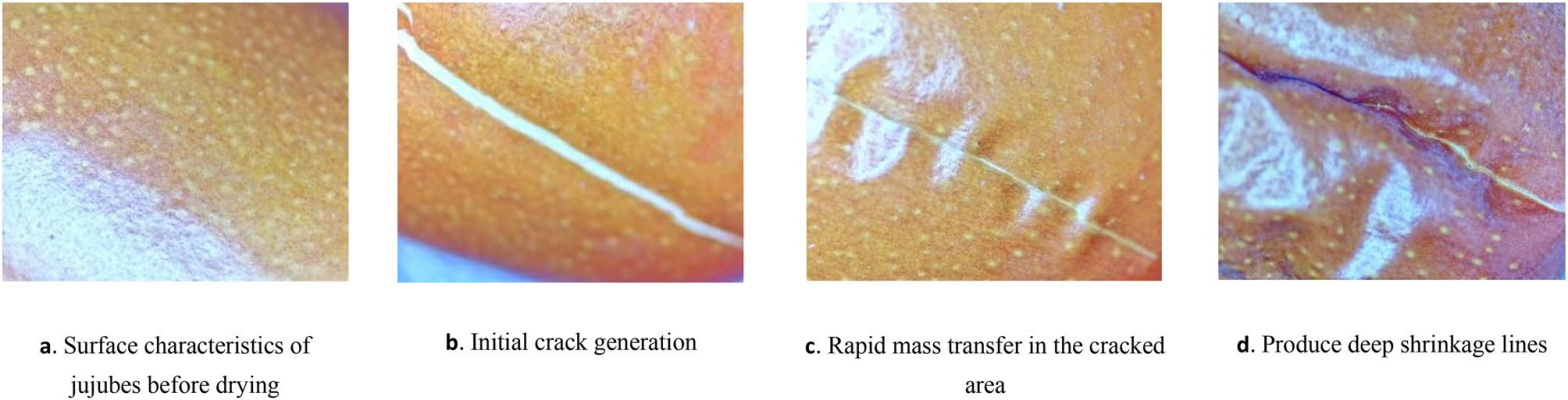
Dynamics of hot air drying cracks in jujubes.

The formation of drying cracks is related to the drying method, moisture content, mechanical properties, and tissue structure of the material^49^. The cracking rate of jujubes during hot air drying continuously decreased with the prolonged picking stage. The early stage of hot air drying is the primary stage in which cracks form, and a higher initial moisture content increases the likelihood of crack formation in the early stage. The cracking rate of jujubes at the end of drying was 16.7%, 13.3%, 11.6%, 5.0%, 0%, and 0% at initial dry base moisture contents of 208%, 158%, 111%, 78%, 63% and 56%, respectively (Table 5). It is believed that hot air drying of jujubes with a high moisture content (S1–S3) heats the internal water in the fruit, which rapidly vaporizes. Figure 6a depicts the condition of fresh jujube before drying. The fruit could not completely discharge the water in time, causing internal expansion and cracking of the peel under stress (Fig. 6b). This phenomenon generally occurs during the accelerated drying phase. Owing to the strong water ability of the crack site to secure the water molecules, the mass transfer rate of the cracked area will be significantly higher than that of the rest of the area (Fig. 6c), resulting in crack diffusion, juice outflow, linear deep shrinkage, and other conditions that impact drying quality (Fig. 6d). Therefore, the drying process parameters, including low temperatures and increased humidity, should be regulated when jujubes have high moisture to limit the heat mass transfer rate of the surface during the early drying phase.

**Table 5:** Changes in the formation of cracks during hot air drying of jujubes at various stages of delayed harvest.

Picking stage	Drying quantity (pieces)	Number of fruit that cracked	Drying crack rate
S1	60	10	0.167
S2	60	8	0.133
S3	60	7	0.116
S4	60	3	0.050
S5	60	0	0.000
S6	60	0	0.000

#### Comprehensive evaluation of the delayed harvest stages

As illustrated in Table 6, the average quality of hot air-dried jujube at each picking stage ranged between 11.29 and 13.25 g. Considering the changes in the nutrient content of fresh fruits at different stages and the actual drying conditions, it is believed that jujubes at the picking stage have a high initial moisture content, an extended hot air drying time, and a rapid heat and mass transfer rate, resulting in the thermal decomposition and sugar precipitation^11^. In contrast, low moisture content impacts the quality of jujube after drying due to nutrient depletion by respiration during the delayed harvest process. Unlike heat-tolerant substances such as sugars, ascorbic acid is highly vulnerable to oxidation and thermal degradation during high-temperature drying; determining ascorbic acid concentrations is better suited to evaluating the quality of dried fruit^50^. In the present investigation, because the weight after drying differed significantly (*P* < 0.05), we determined the mass of ascorbate in a single dried fruit by multiplying the percentage of ascorbate measured by the mass after drying. The initial content of ascorbate decreased with the extension of the picking stage; however, the rate of ascorbate retention increased owing to the reduction in hot air drying time required. Surface water evaporation and chemical reactions such as enzymatic browning, non-enzymatic browning, and caramelization reactions can cause differences in the color of jujubes during high-temperature drying^51^. The color of jujube was compared before and after drying. The results revealed that the brightness L* of jujube in the S1-S3 stage decreased by 34%, 32%, and 21.3% during the different stages, respectively, whereas the brightness L* of jujube in the S4-S6 stage did not change significantly. These data demonstrated that hot air drying potentially influenced the brightness L*, and a higher moisture content resulted in a longer drying time and a higher degree of influence. However, the L* of the S1 stage remained the highest. The red and green value a* improved significantly during the S1 stage, i.e., the green faded and the red colors brightened compared with before drying, and there was no significant change in the other stages. These observations indicated that hot air drying primarily influenced the contents of green compounds on the jujube surface^34^. The blue-yellow value b* of the samples at each picking stage after drying was significantly the highest in S1 and the lowest in S6 (*P* < 0.05), comparable to before drying. Furthermore, there was no significant variation in color at each stage after drying (*P* < 0.05). Rehydration reflects the structural damage of the jujubes after drying. The jujube harvested at the S4–S6 stage rehydrated the fastest after drying, while that picked at the S1–S2 stage rehydrated the slowest. It is considered that the rapid thermal expansion and water-loss shrinkage during the hot air drying of jujube with a high initial moisture content caused more irreversible damage to the tissue structure of fruit and a poor intercellular water storage capacity^52^. The five indicators described above were combined with the drying time and the crack rate to assess jujube quality after hot air drying.

**Table 6:** Statistics on the quality of jujubes after drying at various stages of delayed harvest.

Picking stage	Average weight per fruit after drying g	Color				Ascorbic acid content mg/100 g	Rehydration ratio
		Brightness L*	Red and green values a*	Blue and yellow values b*	Color difference value ΔE		
S1	12.67 ± 0.63ab	33.66 ± 2.36a	17.20 ± 2.54b	27.47 ± 2.02a	–	56.59 ± 1.78e	1.21 ± 0.17c
S2	13.25 ± 0.76a	27.05 ± 1.36b	20.61 ± 1.79a	17.37 ± 1.70b	12.54 ± 1.13a	63.04 ± 2.44d	1.25 ± 0.02c
S3	12.21 ± 0.31bc	27.19 ± 2.02b	21.55 ± 2.13a	17.18 ± 1.53b	12.90 ± 1.58a	82.80 ± 1.05b	1.46 ± 0.15b
S4	11.65 ± 0.61bc	30.40 ± 1.57ab	22.86 ± 2.21a	13.82 ± 0.18c	15.13 ± 2.27a	87.14 ± 1.27a	1.63 ± 0.11a
S5	11.32 ± 0.43c	28.95 ± 2.71b	23.49 ± 1.80a	13.58 ± 1.78c	15.95 ± 1.87a	73.65 ± 3.92c	1.65 ± 0.16a
S6	11.29 ± 0.40c	29.59 ± 1.49b	23.44 ± 1.45a	13.31 ± 1.06c	16.00 ± 2.14a	58.03 ± 2.30e	1.61 ± 0.21a

These five indices, combined with the drying time and crack rate, defined the quality of jujube after drying. A consistency test was performed after constructing a pairwise comparison matrix of factors using the 1–9 ratio scale approach. The weights of ascorbic acid content, rehydration ratio, drying time, crack rate, and color difference were set at 0.3, 0.15, 0.3, 0.15, and 0.1, respectively^53^. The comprehensive scores of hot-air drying quality at the picking stages of S1–S6 were 0.15, 0.27, 0.58, 0.79, 0.73, and 0.59, respectively (Table 7). While the jujubes picked in the earlier stages had a standard coloration and the drying time and crack rate of the jujubes picked in the later stages were lower, the overall quality of the samples picked in the S4-S5 stage remained the highest. Therefore, the Jun jujubes picked at this stage were more suited for drying and processing.

**Table 7:** Results of the comprehensive evaluation of the jujube picking stage.

Picking stage	Normalized Ascorbic Acid Content	Normalized value of compound water ratio	Drying time normalized value	Cracking rate normalized value	Color difference normalized values	Overall rating
S1	0.1722	0.0000	0.0000	0.0000	1.0000	0.15
S2	0.4996	0.0909	0.1875	0.2036	0.2163	0.27
S3	0.9901	0.5682	0.5000	0.3054	0.1938	0.58
S4	1.0000	0.9545	0.7500	0.7006	0.0544	0.79
S5	0.4972	1.0000	0.9375	1.0000	0.0031	0.73
S6	0.0000	0.9091	1.0000	1.0000	0.0000	0.59

## Conclusions

The delayed harvest stage substantially influenced the nutrient and hot air drying characteristics of jujubes, with varied patterns of change for each indicator. The percentages of SS, TS, and RS increased as the picking stage was delayed, while the total weight in individual fruits increased and subsequently decreased until peaking at the S2 stage. There was a distinct drop in the proportion of ascorbic acid and weight within a single fruit at this stage. At the same time, the percentage of TA did not vary considerably. Additionally, the weight of a single fruit decreased, the sugar-acid ratio continually increased, and the fruit tasted sweeter. The brightness of fruit dropped progressively with picking time, and there was no discernible difference beyond the S4 stage. The time required for hot air drying at each stage gradually decreased, but the amount of ascorbic acid in the fruit gradually increased. The level of ascorbic acid in the jujube fruit was the highest after stage S4. Stereomicroscopy examination of the surface cracks of jujube fruits after drying revealed that jujubes picked in the S4-S5 stage cracked less frequently. The trend of change in water rehydration was comparable to the trend of crack generation. The changes in hot air drying and quality characteristics were integrated, and the jujubes picked at the S4 stage were deemed better suited for subsequent hot air drying processing.

## Data Availability

The data presented in this study are available on request from the corresponding author.
